# Potential of Plant Essential Oils and Their Components in Animal Agriculture – *in vitro* Studies on Antibacterial Mode of Action

**DOI:** 10.3389/fvets.2015.00035

**Published:** 2015-09-14

**Authors:** Corliss A. O’Bryan, Sean J. Pendleton, Philip G. Crandall, Steven C. Ricke

**Affiliations:** ^1^Department of Food Science, Center for Food Safety, University of Arkansas, Fayetteville, AR, USA

**Keywords:** essential oils, foodborne pathogens, mode of action, quorum sensing, antibacterial

## Abstract

The antimicrobial activity of essential oils and their components has been recognized for several years. Essential oils are produced as secondary metabolites by many plants and can be distilled from all different portions of plants. The recent emergence of bacteria resistant to multiple antibiotics has spurred research into the use of essential oils as alternatives. Recent research has demonstrated that many of these essential oils have beneficial effects for livestock, including reduction of foodborne pathogens in these animals. Numerous studies have been made into the mode of action of essential oils, and the resulting elucidation of bacterial cell targets has contributed to new perspectives on countering antimicrobial resistance and pathogenicity of these bacteria. In this review, an overview of the current knowledge about the antibacterial mode of action of essential oils and their constituents is provided.

## Introduction

Plant materials including flowers, roots, bark, leaves, seeds, peel, fruits, and wood can be used to extract aromatic and volatile liquids known as essential oils (EOs) (1–3). These EOs have a long history of use for medical purposes, in perfumes and cosmetics, and as herbs and spices for foods. EOs are considered to be secondary metabolites in plants; secondary metabolites are organic compounds that are not directly involved in the normal growth, development, or reproduction of the plant (4). These secondary metabolites are often involved in plant defense and thus may possess antimicrobial properties (4, 5). The first experiment to determine the bactericidal properties of EOs is said to have been carried out by de la Croix in 1881 (6). In more recent years, many EOs, or their components, have been shown to possess broad-range antibacterial properties (7).

Increased resistance to infectious diseases, including parasitic infections such as coccidiosis, has been noted when plant phytonutrients were fed to animals. For instance, Lee et al. (8) found that feeding plum powder to laying hens increased their immune response as well as conferring immunity to coccidiosis. Lillehoj et al. (9) determined the effects of feeding capsicum oleoresin or cinnamaldehyde on the global gene expression profiles of broilers. Capsicum oleoresin induced gene changes in genes associated with metabolism and immunity, whereas cinnamaldehyde affected genes related to antigen presentation, humoral immune response, and inflammatory disease. Feeding of these compounds also protected the birds against infection with live coccidiosis parasites. Mathlouthi et al. (10) found that oregano or rosemary EOs had different antimicrobial effects *in vitro* against pathogenic and non-pathogenic bacteria but had the same growth promoting effects as avilamycin when added to broiler diets. Authors speculated that the *in vivo* growth promotion effects were due to ecological changes in the bacterial gut flora rather than antibacterial effects against a single bacterial genus and species. Betancourt et al. (11) confirmed a shift in gut flora in the foregut but not ceca and colon in broilers fed oregano EOs during a 42-day grow out period.

Alali et al. (12) tested a mixture of carvacrol, thymol, eucalyptol, and lemon for the ability to prevent colonization and shedding in broilers intentionally fed *Salmonella* Heidelberg. They determined that feeding 0.05% (v/v) of the EO mixture significantly reduced the colonization of the crops of challenged birds as well as lowering feed conversion and improving weight gain in the birds. However, cecal colonization and shedding were not significantly decreased. Cerisuelo et al. (13) fed an EO mixture composed of cinnamaldehyde and thymol to broilers, either with or without butyric acid. They determined that the EO blend reduced cecal numbers of *Salmonella*, especially when combined with butyric acid, as compared to control feed. Ricke et al. (14) provide an overview of the anti-*Salmonella* effects of EOs in agriculture.

Benchaar et al. (15) investigated the effects of EOs *in vitro* rumen microbial fermentation. They determined that only the phenolic compounds, carvacrol, thymol, and eugenol affected ruminal fermentation, relative to the control, increasing pH and butyrate and decreasing propionate, indicating antibacterial activity which was not nutritionally beneficial. Callaway et al. (16) studied the *in vitro* effects of orange peel and orange pulp, both sources of EOs, against *Escherichia coli* O157:H7 and *Salmonella typhimurium* in rumen fluid. Growth of both pathogens was reduced by addition of 0.002 g/ml of orange pulp or orange peel. Callaway et al. (17) were able to demonstrate that the orange peel products when fed to experimentally inoculated sheep reduced *S*. *typhimurium* populations in the gut, with a significant reduction reached in the ceca.

The antimicrobial properties of EOs are a recent focus for agricultural applications because of a desire on the part of many consumers to reduce the use of “hazardous or unnatural chemicals” in their food (18–20). Although there are many studies on the antimicrobial activities of EOs, few take the next step and determine the mode of action of these compounds. However, application of EOs as antibacterial substances for food animals or as food preservatives requires detailed knowledge about their properties, including the mode of action. The purpose of this review is to provide an overview of current knowledge about the antimicrobial mode of action of EOs and their constituents.

## Effects on Cell Wall and Membrane

Antimicrobial activity of EOs is strongly linked to their hydrophobicity (21–28). The cell walls of Gram-positive bacteria are made up predominantly of peptidoglycan linked with other molecules such as proteins or teichoic acid (29). On the other hand, Gram-negative bacteria possess an outer membrane of containing hydrophilic lipopolysaccharides (LPS), which creates a barrier toward hydrophobic compounds such as those found in EOs (30, 31). Gram-negative bacteria are thus considered to be less susceptible to the effects of EOs than Gram-positive bacteria (32). However, the hydrophobic constituents of EOs are able to gain access to the periplasm of Gram-negative bacteria through the porin proteins of their outer membrane (24), through which they can slowly travel (33) See Table 1 for an overview of the bacterial targets of select EOs and their constituents.

**Table 1:** **Target of antibacterial action of some essential oils**.

Target	Essential oil or component	Reference
Cell wall or membrane	Carvacrol	Helander et al. (24), Ultee et al. (28, 34), Fitzgerald et al. (37), Xu et al. (48)
	Cinnamon	Bouhdid et al. (40)
	Cinnamaldehyde	Gill and Holley (57)
	Cymene	Ultee et al. (28)
	Eugenol	Walsh et al. (36)
	Farnesol	Inoue et al. (38)
	Nerolidol	Inoue et al. (38)
	Oregano	Bouhdid et al. (43), De Souza et al. (53)
	Plaunotol	Inoue et al. (38)
	Rosemary	De Souza et al. (53)
	Tea tree oil	Cox et al. (26)
	Thymol	Helander et al. (24), Walsh et al. (36), Xu et al. (48)
	Vanillin	Fitzgerald et al. (37)
Respiration	Cinnamon	Bouhdid et al. (40)
	Tea tree oil	Cox et al. (26)
	Vanillin	Fitzgerald et al. (37)
Quorum sensing	Clove	Khan et al. (64)
	Geranium	Szabo et al. (65)
	Lavender	Szabo et al. (65)
	Oregano	Alvarez et al. (66)
	Rose	Szabo et al. (65)
	Rosemary	Szabo et al. (65)

### Potassium leakage from cells

The leakage of potassium into the extracellular space is considered an indicator for an increase in membrane permeability and ultimate loss of viability for the cell. This particular result was observed in multiple studies (22, 26, 34–40). Carvacrol is a fraction of many EOs and is the major component of oregano EO, which consists of 60–74% carvacrol and thyme EO which consists of 45% carvacrol (41, 42). Ultee et al. (34) examined carvacrol for its antimicrobial properties against *Bacillus cereus* and determined that, at a concentration of 0.15 ml/l, carvacrol caused an immediate decrease in intracellular potassium and an increase in the extracellular potassium. Fitzgerald et al. (37) studied the effects of carvacrol and vanillin on *E. coli*, *Lactobacillus plantarum*, and *Listeria innocua*. At concentrations of 7.6 and 0.496 ml/l of vanillin and carvacrol, respectively, they saw that intracellular potassium levels decreased and the extracellular potassium levels increased in all organisms. Both thymol and eugenol were able to increase extracellular potassium at concentrations of 0.50 ml/l in *E. coli* and *Staphylococcus aureus* (36). Tea tree oil at 2.50 ml/l caused the release of 100% of the total cellular potassium in *E. coli* within 30 min, but only approximately 20% was released by *S. aureus* in the same time (26), which is in contrast to the reported greater susceptibility of Gram-positives (32) and illustrates the great diversity in efficacy of EOs. Bouhdid et al. (43) demonstrated that oregano EO caused potassium leakage in both *Pseudomonas aeruginosa* and *S. aureus*. Oil-treated cells were examined by transmission electron microscopy (TEM), which revealed mesosome-like structures, coagulated cytoplasmic material, and intracellular material outside the cell; these effects were more noticeable in *P. aeruginosa* compared with *S. aureus* (43). Bouhdid et al. (40) determined the effects of EO of *Cinnamomum verum* on *P. aeruginosa* and *S. aureus* and discovered that a concentration of 1.25 ml/l was able to increase extracellular potassium levels in both bacteria. Inoue et al. (38) tested the terpene alcohols farnesol, nerolidol, and plaunotol for their antimicrobial effects on *S. aureus*. All three compounds increased the extracellular potassium levels when applied to *S. aureus* at a concentration of 0.020 ml/l. Togashi et al. (39) found that when geraniol was added to farnesol in a ratio of 0.010:0.005 ml/l, the ability of farnesol to cause potassium leakage was enhanced, while geranylgeraniol inhibited the potassium leakage activity of farnesol. Bajpai et al. (44) used scanning electron micrographs of *B. cereus* and *E. coli* to demonstrate that cells treated with *Ginkgo biloba* EO (250 and 500 μg/ml, respectively) had disruption of cell membranes and swelling of the cells which led to leakage of potassium from the cells. The studies discussed in this section indicate that these EOs and components act in a similar fashion on the membrane to cause the bacterial cell to lose the ability to regulate potassium transfer across the membrane, leading to an outpouring of potassium from the cell and a subsequent loss of viability.

### Leakage of other cell components

The release of other cellular components has also been an object of study for those seeking to determine microbial action of EOs. Carboxyfluorescein diacetate is commonly used as a probe for viable cells (45, 46). Carboxyfluorescein diacetate was used to stain live cells of *L. innocua* which were then exposed to the EOs of *Cymbopogon citratus*, *Ocimum gratissimum*, or *Thymus vulgaris* (47). Loss of fluorescence was taken to be indicative of leakage of carboxyfluorescein from within the cell and thus a measure of the disruption of the cell membrane. EOs of *C. citratus* (7.80 ml/l), *O. gratissimum* (5.0 ml/l), and *T. vulgaris* (4.30 ml/l) were adjusted to a concentration of 1.04 × 10^4^ arbitrary units (AUs) for treatment; AUs were determined by the formula AU ml^−1^ = mean diameter (mm) of the minimal inhibition zone × dilution factor × 50. All EOs were shown to cause a decrease in fluorescence as compared to an unexposed control (47). Xu et al. (48) also examined the leakage of carboxyfluorescein by *E. coli* after exposure to components of thyme EO, carvacrol and thymol, at different concentrations (0.10 and 0.20 ml/l). They found that both carvacrol and thymol caused an increase in carboxyfluorescein released by *E. coli* cells. Trombetta et al. (32) assessed the release of carboxyfluorescein from large unilamellar vesicles (LUVs) that were exposed to (+) menthol, thymol, and linalyl acetate at several concentrations (0.10, 0.25, and 0.50 ml/l). They concluded that (+) menthol and thymol were both effective in causing the release of carboxyfluorescein from the LUVs, but linalyl acetate was only slightly effective. Helander et al. (24) researched the release of fatty acids from *E. coli* cells treated with thymol, carvacrol, (+)-carvone, or *trans*-cinnamaldehyde. Thymol and carvacrol were shown to release a significant amount of fatty acids into the supernatant, while (+)-carvone and *trans*-cinnamaldehyde were ineffective at releasing fatty acids. The results produced by Helander et al. (24) demonstrated that thymol and carvacrol actually break down the cell membrane. Proteins absorb light at 280 nm; therefore, release of 280 nm materials was considered indicative of macromolecular leakage by the cell; *E. coli* and *S. aureus* exposed to tea tree oil were found to leak 280 nm absorbing material, but at levels lower than potassium leakage (35). Nucleic acids, on the other hand, absorb light at 260 nm, and the release of 260 nm material from bacterial cells would be indicative of macromolecular leakage by the cell. Ifesan et al. (49); Carson et al. (50), and Oussalah et al. (51) all examined the release of 260 nm absorbing material. Strains of *S. aureus* exposed to a crude extract of a perennial herb, *Eleutherine americana*, had a peak release of 260 nm absorbing materials after 8 h of exposure to 2.50–10.0 ml/l of the extract (49). After 22 h of exposure, the absorbance decreased from the levels seen after 8 h, most likely due to denaturation of the materials, which caused them to become unreactive to the light at 260 nm (49). A significant amount of 260 nm material was released by *S. aureus* cells after an hour of exposure to tea tree oil or its components (50). Oussalah et al. (52) examined Spanish oregano, Chinese cinnamon, and savory EOs against *E. coli* O157:H7 and *Listeria monocytogenes*. They found that at 0.25 ml/l each EO was able to cause an increase in the release of 260 nm absorbing material. A study investigating the effects of the EOs of oregano and rosemary on *Pseudomonas fluorescens* found that cell material was released into the growth medium immediately after contact with either EO singly or in combination (53). Electron microscopy of exposed cells revealed alteration in the cell wall structure, rupture of the plasma membrane, shrinking of the cells, condensation of the cytoplasmic content, and leakage of the intracellular material with a 2 h contact time (53). Confocal scanning laser microscopy revealed increased cell membrane permeability, resulting in cell death after exposure times of only 15 min (53). These studies indicate that EOs and components are able to cause macromolecular permeability in a variety of bacteria.

### Uptake of substances

The cell membrane helps cells regulate what enters and exits the cell. When ions are no longer being regulated, it indicates that at the very least small pores have formed in the cell membrane. When larger molecules, such as propidium iodide (PI) or *N-*phenyl-l-napthylamine (NPN), enter the cell without regulation it indicates much larger pores have formed in the membrane and a much higher probability of cell death. Helander et al. (24) examined the uptake of NPN by *E. coli* and *S. typhimurium* after exposure to thymol, carvacrol, (+)-carvone, and *trans*-cinnamaldehyde. Thymol and carvacrol significantly increased the uptake of NPN by both bacteria, while (+)-carvone and *trans*-cinnamaldehyde did not allow uptake. These findings demonstrate that (+)-carvone and *trans*-cinnamaldehyde are unable to form pores large enough for NPN, unlike thymol and carvacrol. *Trans*-cinnamaldehyde has a similar minimal inhibitory concentration (MIC) to carvacrol and thymol, according to Helander’s study, but it does not function as a large pore former like carvacrol or thymol. Therefore, there must be another mode of action for *trans*-cinnamaldehyde. Fisher and Phillips (54) also investigated NPN uptake by *Enterococcus faecium* and *Enterococcus faecalis* after exposure to a citrus oil blend. They found that the oil blend was able to increase the NPN uptake over twofold, indicating that the blend is able to cause large pore formation in the bacteria. They also examined the cells using TEM and reported morphological changes in the cells including a loss of distinction of the membrane after being in contact with either the EO or its vapor. Cells that had been in direct contact with the EO appeared to have vacuoles which the authors speculated contained the EO within them. Nearly 100% of *E. coli* cells exhibited increased PI uptake after exposure to tea tree oil, while only around 10% of *S. aureus* cells expressed increased PI uptake. (35). Cox’s results indicate that there is a possible difference in the effects of tea tree oil on Gram-negative and Gram-positive bacteria. Similarly, Bouhdid et al. (40) determined that cinnamon EO treatment made *P. aeruginosa* much more susceptible, in terms of PI uptake, than *S. aureus*. Fitzgerald et al. (37) exposed *E. coli*, *L. plantarum*, and *L. innocua* to vanillin and measured subsequent PI uptake; *E. coli* exhibited membrane damage after 15 and 60 min of exposure, but appeared to recover overnight, as the percentage of membrane damage decreased from that seen after 60 min. However, *L. plantarum* exhibited slight damage after 15 and 60 min, but rather than recover overnight as *E. coli* did, damage increased (37). *L. innocua* showed little susceptibility to vanillin. Fitzgerald’s results indicate that vanillin does not have a Gram-negative or Gram-positive bias, such as observed with tea tree oil or cinnamon EO, although further studies with tea tree oil and cinnamon EO need to be carried out to determine if they are more effective at large pore formation in Gram-negative bacteria. Based on these studies, large pore formation appears to be a mode of action employed by various EOs against a range of bacteria.

### Effects on membrane potential

Membrane potential is used by the cell to perform actions necessary for life, such as synthesis of enzymes, nucleic acids, polysaccharides, and other cell components, for cell maintenance and repair of damage, for motility, and for numerous other processes (55), and decrease in this membrane potential is indicative of damage to the cell membrane. Bouhdid et al. (40) used bis-oxonol dye to stain depolarized *P. aeruginosa* and *S. aureus* cells. They found that cinnamon EO at MIC levels was able to decrease the membrane potential of *P. aeruginosa*, but not *S. aureus*. Using 3,3′-dipropylthiacarbocyanide iodide (DiSC_3_5) to monitor membrane potential, Veldhuizen et al. (56) exposed *S. aureus* cells to carvacrol, *o*-cresol, or 2-amino-*p*-cymene at MIC concentrations which were all found to decrease the membrane potential of the cells within seconds of contact. Ultee et al. (28) also employed DiSC_3_5 to look at the disruption of membrane potential of *B. cereus* by carvacrol and cymene. Carvacrol was seen to immediately decrease the membrane potential at concentrations above 0.037 ml/l, while cymene was able to decrease the membrane potential to a lesser extent at a maximum concentration of 0.067 ml/l. Cymene lacks the hydroxyl group that is present on carvacrol, indicating that the hydroxyl group plays an important role in the effect of carvacrol on the membrane potential. Fisher and Phillips (54) also monitored membrane potential with DiSC_3_5 in *E. faecalis* and *E. faecium* after exposure to a 1:1 blend (20.0 ml/l each) of orange and bergamot EOs. They found that the blend was able to dissipate the membrane potential within seconds of contact with the cells. These studies show that when an EO affects the membrane potential of a cell it is an almost immediate reaction. Loss of membrane potential is adverse to cell survival, but could be a consequence of membrane disruption in the case of carvacrol.

### Effects on ATP: Leakage from cells and usage by cells

Another target for research on the mode of action of EOs has been to examine the levels of ATP both inside and outside bacterial cells. Ultee et al. (28) determined that cymene, a plant aromatic compound found in cumin and thyme, was unable to cause ATP levels to rise in the extracellular environment or decrease in the intracellular environment. However, carvacrol was able to eliminate the internal ATP of *B. cereus* cells within 20 min of contact at a concentration of 0.150 ml/l (34). Fisher and Phillips (54) utilized an EO blend consisting of a 1:1 mixture of sweet orange and bergamot to treat *E. faecalis* and *E. faecium* and determined that the blend was able to completely dissipate internal ATP in within 10 min of contact. Oussalah et al. (51) examined the effects of Spanish oregano, Chinese cinnamon, and savory EO on the ATP of *E. coli* O157:H7 and *L. monocytogenes*. Savory EO was able to increase extracellular ATP levels in both bacteria, while Spanish oregano was only able to increase extracellular ATP in *E. coli* O157:H7 and Chinese cinnamon was not able to increase extracellular ATP in either organism. Helander et al. (24) looked at the effects of carvacrol, thymol, (+)-carvone, and *trans*-cinnamaldehyde on the ATP of *E. coli* cells. Carvacrol and thymol were both found to decrease intracellular ATP over a 20-min period, and slightly increase external ATP, but (+)-carvone and *trans-*cinnamaldehyde did not affect the ATP of the cells, internally or externally. Fitzgerald et al. (37) looked at the effect of vanillin and carvacrol on ATP of *E. coli*, *L. plantarum*, and *L. innocua*. Vanillin was found to be ineffective at reducing intracellular ATP or increasing extracellular ATP in any bacteria. Carvacrol was very effective at decreasing all bacteria’s internal ATP, while only increasing external ATP in *E. coli* and *L. innocua*. The increase in extracellular ATP concentration in the presence of some EOs is of course due to intracellular ATP release, most likely as a consequence of envelope damage induced by the oils.

Some EOs and components, such as cinnamaldehyde, directly target the production of ATP in the cell. Gill and Holley (57) investigated ATP generation in *Lactobacillus sakei* and *L. monocytogenes*. Both bacteria were treated with eugenol, and *L. monocytogenes* was additionally treated with cinnamaldehyde. Eugenol was found to inhibit ATP production but was unable to decrease intracellular ATP. Cinnamaldehyde was found to both inhibit production of ATP and decrease intracellular ATP. These results were determined by adding the EOs to the bacteria before or after the addition of 0.25% glucose to energize the cell. Gill and Holley (57) propose that the mode of action for eugenol relies on inhibiting the cell from using glucose due to its effectiveness when added prior to glucose, but not when added after glucose. Cinnamaldehyde reduced ATP levels when added either prior to or after glucose, which led Gill and Holley (57) to hypothesize that there may be more than one plausible mode of action. Eugenol, carvacrol, and cinnamaldehyde have been found to inhibit the membrane-bound ATPase activity of some bacteria. There are several enzymes with ATPase activity that are associated with bacterial membranes, including one that is involved in ATP generation and cellular pH regulation (58). Gill and Holley (59) hypothesized that ATPase inhibition plays a significant role in reducing the growth rate of pathogens at sublethal concentrations of the EO.

### Effects on pH gradient

The ability to maintain a pH gradient is necessary for cell survival and thus studying the internal pH or the pH gradient helps to determine if a cell has been severely damaged. Oussalah et al. (51) and Lambert et al. (22) both examined the internal pH of bacteria exposed to EOs. Oussalah et al. (51) found that Spanish oregano, Chinese cinnamon, and Savory EOs were effective at decreasing the internal pH of *E. coli* O157:H7 and *L. monocytogenes*. Lambert et al. (22) determined that oregano EO increased the rate of change in internal pH for *S. aureus* compared with an untreated control, indicating a lack of ability by the cell to maintain the pH gradient. Sikkema et al. (58) tested liposomes that had been reconstituted with beef heart mitochondrial cytochrome c oxidase, in order to give them a proton motive force, with various cyclic hydrocarbons (benzene, toluene, ethylbenzene, *o*-xylene, and others) and found that all of them dissipated the pH gradient at concentrations related to their membrane partition coefficient. Fisher and Phillips (54) employed their 1:1 blend of orange and bergamot EOs (20.0 ml/l of each) to determine that the blend was able to completely eliminate the pH gradients of *E. faecium* and *E. faecalis*. Fitzgerald et al. (37) treated *L. plantarum* with vanillin and determined that 15.2 ml/l of vanillin was able to dissipate the pH gradient. Ultee et al. (28, 34) ascertained that 0.15 ml/l carvacrol completely dissipated the pH gradient across the cytoplasmic membrane of *B. cereus*, while 0.268 ml/l of cymene was ineffective. These studies indicate that many oils act to disrupt the membrane in such a way as to make the cell unable to maintain a pH gradient which is essential for generation of a proton motive force, and thus life.

## Effect on Respiration

Respiration is an integral part of aerobic metabolism with the potential to be a target for antimicrobial EOs, and several researchers have examined the effects of EOs on respiration to help elicit their mode of action. Reduction of 5-cyano-2,3-ditolyl tetrazolium chloride (CTC) is an indication of respiratory enzyme activity; Bouhdid et al. (40) determined that in the presence of cinnamon EO <6% of *P. aeruginosa* cells were able to reduce CTC after a 30-min exposure to the MIC of the oil (1.25 ml/l). However, *S. aureus* was found to be more resistant as approximately 10% of the cells were able to reduce CTC after 60 min of exposure to the MIC (1.25 ml/l) and 1.5× MIC (1.87 ml/l) of the oil. Cox et al. (26) examined the oxygen consumption of *E. coli* and *S. aureus* in the presence of tea tree oil at different concentrations; 5.0 ml/l of tea tree oil reduced the oxygen consumption of *E. coli* by 100% and 10.0 ml/l reduced the oxygen consumption of *S. aureus* by 60%. Fitzgerald et al. (37) determined oxygen consumption in *L. innocua* and *E. coli* when exposed to various concentrations of vanillin (0–6.09 ml/l) and discovered that oxygen consumption was reduced by 19 and 52% in *E. coli* and *L. innocua*, respectively, when exposed to 6.09 ml/l of vanillin. These results indicate that some EOs disrupt the ability of bacterial cells to perform oxidation reduction reactions and could be a potential mode of action for the oil.

## Effect on Quorum Sensing

Cell-to-cell communication among bacteria [quorum sensing (QS)] is accomplished using small signaling molecules produced by the bacteria and is utilized by the bacteria to evaluate their external environment and their internal physiological status (60). The signaling molecules are in general known as autoinducers; the Gram-negative bacteria use acyl homoserine lactones, whereas the Gram-positive bacteria use modified oligopeptides as signaling molecules (61). QS is involved in biofilm production, motility, swarming, stress resistance, and virulence (61). Researchers have begun to target QS as a means to reduce antimicrobial resistance and food spoilage (62). Additionally, QS is thought to allow pathogens to minimize host immune responses by delaying the production of virulence factors until a large enough bacterial population is present to overwhelm host defense mechanisms and establish infection (63).

Khan et al. (64) screened 21 EOs for anti-QS activity using biosensor strains, *Chromobacterium violaceum* CV12472 and CVO26. They determined that clove oil demonstrated the most anti-QS activity on both wild and mutant strains followed in activity by cinnamon, lavender, and peppermint oils. The effect of clove oil on pigment production, which is QS controlled, was found to be concentration dependent. At sub-MICs of clove oil, there was a 78.4% reduction of pigment production compared with the control and up to a 78% reduction in swarming motility over the control (64). Szabo et al. (65) performed similar studies with a wider range of EOs and established that rose, geranium, lavender, and rosemary oils were the most potent QS inhibitors of the compounds they tested. Eucalyptus and citrus oils moderately reduced pigment production by CV026, while chamomile, orange, and juniper oils were ineffective. Alvarez et al. (66) researched the use of oregano EO in edible films and the effect on QS as well as the antibacterial activity against several pathogens. Pectin films incorporated with 36.10 and 25.90 ml/l of oregano EO inhibited growth of *E. coli* O157:H7, *Salmonella* Cholerasuis, *S. aureus*, and *L. monocytogenes*. All concentrations of oregano EO (0.015, 0.0312, 0.0625, and 0.125 ml/l) and pectin-EO films (15.70, 25.90, and 36.10 ml/l) showed a significant anti-QS activity. They also determined that the pectin-EO films reduced total coliforms, yeast, and molds on shrimp and cucumber slices stored at 4°C for 15 days. Thus, EOs could be used to explicitly target QS in a novel antimicrobial strategy.

## Effect of Compound Structure on Antimicrobial Activity

Many researchers have examined the structures of EOs themselves to seek to determine what allows these compounds to affect the various cellular activities of bacteria. Sikkema et al. (58) examined how biological membranes react to cyclic hydrocarbons and determined that the hydrocarbons accumulate in the interior of the membrane causing it to swell. The amount to which a particular hydrocarbon can accumulate in the membrane was found to be directly related to its membrane partition coefficient. Sikkema et al. (58) proposed that the accumulation of these compounds in the membrane result in changes to the membrane structure and function, thus leading to inhibition or cell death.

Carvacrol is an EO that has been shown to have high antibacterial activity and therefore it has been the focus or benchmark for many studies, some of which have studied what it is about the structure of carvacrol that makes it antimicrobial. Carvacrol is a cyclic hydrocarbon which, according to Sikkema et al. (58), allows it to accumulate in the membrane. It also has a hydroxyl group on the ring, which Ultee et al. (28) have proposed gives carvacrol its activity. Ultee et al. (28) also studied thymol, menthol, carvacrol methyl ether, and cymene in addition to carvacrol. These compounds possess similar structures to carvacrol that only differ in one or two ways. Thymol has the same chemical formula as carvacrol, but the hydroxyl group is in the meta position rather than the ortho position. Menthol lacks the benzene ring and instead has a cyclic hexane ring. Carvacrol methyl ether has the hydroxyl group replaced with a methyl ether group. Finally, cymene completely lacks a hydroxyl group. Ultee et al. (28) noted that cymene and carvacrol methyl ether lacked any antibacterial activity. Menthol showed slight inhibitory activity at a concentration of 0.320 ml/l for up to 5 h, but after 24 h, the bacteria had rebounded to levels seen with only a 0.016 ml/l concentration of menthol, which showed no inhibition of the bacteria. Thymol showed a very similar pattern of activity to that of carvacrol, with concentrations above 0.113 ml/l completely inhibitory to the bacteria (28).

Veldhuizen et al. (56) achieved similar results when they examined *o*-cresol, 3-isoporpylphenol, 2-amino-*p*-cymene, *p*-cymene, and 3,4-dimethylcumene in comparison to carvacrol. They found that *p*-cymene and 3,4-methylcumene exhibited no antibacterial activity. In 3,4-methylcumene, there is a methyl group in the place that the hydroxyl group of carvacrol occupies, indicating that the hydroxyl group is an important component of the antibacterial activity. The compound 3-isopropylphenol, which lacks the methyl group on the ring, was found to have an MIC approximately 1.5 times that of carvacrol, while *o*-cresol, which lacks the isopropyl group of carvacrol, was found to have an MIC approximately two times that of carvacrol. The MIC of these two carvacrol-related compounds indicates that the R groups do play a small role in the antibacterial activity of carvacrol, though not as much as the hydroxyl group. The compound 2-amino-*p*-cymene has an amino group in the same position as the hydroxyl group of carvacrol, but the rest of the structure remains the same. The MICs of 2-amino-*p*-cymene were approximately 0.450–0.60 ml/l higher than that of carvacrol and had the highest MIC of all antibacterially active compounds tested. Since 2-amino-*p*-cymene shows antibacterial activity, it seems that the hydroxyl group is not essential for antimicrobial activity, although it appears to enhance activity considerably.

Ben Arfa et al. (67) also examined how the structure of carvacrol affects its antibacterial activity. They compared carvacrol with carvacrol methyl ether, carvacrol acetate, eugenol, and menthol. Carvacrol methyl ether and carvacrol acetate were not found to display any antibacterial activity; both of these compounds have substitutions at the hydroxyl group of carvacrol respective of their names, indicating that these groups cannot convey the same activity as the hydroxyl group of carvacrol. Eugenol, which is another aromatic compound like carvacrol, has a hydroxyl group and an ether group which are para and ortho, respectively, to a three-carbon chain that ends in a double bond. Eugenol was found to exhibit a lower antibacterial activity than carvacrol, but was not inactive like carvacrol acetate and carvacrol methyl ether. Ben Arfa et al. (67) only determined MICs for their compounds, so it is difficult to say if the antibacterial action of eugenol is the same as carvacrol, although it can be said that not all antibacterial EO compounds must have a structure similar to that of carvacrol in order to possess activity. Similar to Ultee et al. (28), Ben Arfa et al. (67) found menthol to possess very little antibacterial activity, which supports the theory that the benzene ring is important for antibacterial activity, but only for those compounds that possess a hydroxyl group or an amino group, as was the case with 2-amino-*p*-cymene in the study by Veldhuizen et al. (56).

Gill and Holley (57) studied the two aromatic compounds eugenol and *trans*-cinnamaldehyde. Both compounds were found to exhibit antibacterial activity, although eugenol had a lower MIC. *Trans*-cinnamaldehyde, which contains an aromatic ring with a three-carbon aldehyde chain containing a double bond at the second position, was able to decrease internal ATP of energized cells and inhibit ATP increases of non-energized cells of *L. monocytogenes*. Eugenol, which was described earlier, was able to inhibit ATP increases of non-energized cells, but was unable to reduce ATP levels of energized cells. Their results indicate that *trans*-cinnamaldehyde could be acting via membrane disruption without the need of the carvacrol hydroxyl group. It is difficult to compare carvacrol and *trans*-cinnamaldehyde since they were not used in the same study, but if one extrapolates the results from Gill and Holley (57) and Ben Arfa et al. (67), one can make the assumption that *trans*-cinnamaldehyde has lower antibacterial activity than carvacrol. This is due to the fact that *trans*-cinnamaldehyde was found to have lower antibacterial activity than eugenol and that eugenol was found to have lower antibacterial activity than carvacrol. Therefore, *trans*-cinnamaldehyde probably causes membrane disruption to a lesser extent than carvacrol due to its lack of a hydroxyl group. Bouhdid et al. (40) examined cinnamon EO, which is mainly composed of *trans*-cinnamaldehyde, and found that the EO caused leakage of potassium ions from *P. aeruginosa* and *S. aureus*, which furthers the idea that *trans*-cinnamaldehyde is a membrane disruptor. Helander et al. (24), however, found that *trans*-cinnamaldehyde was unable to induce ATP leakage. This contradictory result is most likely due to the concentration difference used in the two studies; Helander et al. (24) used 0.264 ml/l, whereas Gill and Holley (57) used 5.29 ml/l.

## Conclusion

Several plant-derived EOs are effective as natural alternatives to synthetic food additives, particularly as antimicrobial agents. One of the main actions of EOs is to affect the permeability of cell membranes leading to loss of cellular components or influx of other substances into the cell. New studies on the effects of EOs on the QS systems of foodborne and other pathogens offer an opportunity for the development of natural food antimicrobials that could not only be bactericidal but could also lessen pathogenic effects.

## Conflict of Interest Statement

The authors declare that this review was prepared in the absence of any commercial or financial relationships that could be construed as a potential conflict of interest.
